# Expression and purification of untagged GlnK proteins from actinobacteria 

**DOI:** 10.17179/excli2017-394

**Published:** 2017-06-27

**Authors:** Edileusa C.M. Gerhardt, Vivian R. Moure, Andrey W. Souza, Fabio O. Pedrosa, Emanuel M. Souza, Lautaro Diacovich, Hugo Gramajo, Luciano F. Huergo

**Affiliations:** 1 Departamento de Bioquímica e Biologia Molecular, UFPR, Curitiba, Brazil; 2 Instituto de Biologia Molecular y Celular de Rosario (IBR-CONICET), Facultad de Ciencias Bioquimicas y Farmaceuticas, Universidad Nacional de Rosario, Rosario, Argentina; 3 Setor Litoral, UFPR, Matinhos, Brazil

**Keywords:** PII, GlnK, CD analysis, actinobacteria

## Abstract

The PII protein family constitutes one of the most conserved and well distributed family of signal transduction proteins in nature. These proteins play key roles in nitrogen and carbon metabolism. PII function has been well documented in Gram-negative bacteria. However, there are very few reports describing the *in vitro *properties and function of PII derived from Gram-positive bacteria. Here we present the heterologous expression and efficient purification protocols for untagged PII from three Actinobacteria of medical and biotechnological interest namely: *Mycobacterium tuberculosis, Rhodococcus jostii *and *Streptomyces coelicolor. *Circular dichroism and gel filtration analysis supported that the purified proteins are correctly folded. The purification protocol described here will facilitate biochemical studies and help to uncover the biochemical functions of PII proteins in Actinobacteria.

## Introduction

Microorganisms have to face a myriad of nutritional conditions through-out their life cycles. The presence of sensory proteins orchestrating a rapid metabolic reprogramming is vital for fitness. The PII protein family constitutes one of the most ancient, widespread and multifunctional family of sensory proteins in nature. They are present in most Bacteria, Archaea and also in the chloroplast of algae and plants (Huergo et al., 2013[10]; Forchhammer and Luddecke, 2016[7]). 

PII proteins are homotrimers which have been firstly described as master regulators of nitrogen metabolism in Gram-negative bacteria controlling ammonium assimilation (Ninfa and Jiang, 2005[19]) and nitrogen fixation (Huergo and Dixon, 2015[11]). Regulation of nitrogen metabolism by PII is also well documented in Archaea, cyanobacteria and plants (Leigh and Dodsworth, 2007[14]; Forchhammer and Luddecke, 2016[7]). A novel conserved facet of PII is its ability to regulate fatty acid metabolism by acting as a dissociable regulatory subunit of acetyl-CoA carboxylase in bacteria and plants (Feria Bourrellier et al., 2010[6]; Gerhardt et al., 2015[8]; Rodrigues et al., 2014[20]). Other sub-families of PII-related signaling proteins have been described such as the cyclic-di-AMP binding proteins PstA and DarA in Gram-positive bacteria (Choi et al., 2015[5]; Muller et al., 2015[18]; Gundlach et al., 2015[9]) and the bicarbonate sensory PII-like protein in autotrophic bacteria (Wheatley et al., 2016[23]).

Canonical PII proteins act in signal transduction due to their ability to bind important allosteric modulators such as 2-oxoglutarate, ATP and ADP. The interaction between these effectors and PII alters the protein structure thereby affecting the interaction between PII and various target proteins (Huergo et al., 2013[10]; Huergo and Dixon, 2015[11]). PII proteins from plants are also allosteric modulated by glutamine (Chellamuthu et al., 2014[4]). In proteobacteria, glutamine also affects PII activity, however, in this case, glutamine allosteric binds to the bifunctional uridylyl-transferase-removing enzyme GlnD which, in turn, regulates PII function by reversible uridylylation (Jiang et al., 1998[12]). In Actinobacteria PII proteins are regulated by reversible adenylylation rather than uridylylation (Merrick, 2014[16]). The presence of multiple PII like proteins is common in Gram-negative bacteria; however, Gram-positive Actinobacteria usually encode only one type of PII protein which has been named GlnK given its genetic linkage to the *amtB *gene encoding the ammonium transporter protein (Sant'Anna et al., 2009[21]).

Despite the wealth of biochemical data describing PII function in Gram-negative bacteria, Archaea and plants much less is known about PII function at biochemical level in Gram-positive Actinobacteria. It has been established that Gram-positive PII proteins (GlnK) interact and regulate the activity of the transcriptional factors GlnR in *Streptococcus mutans *(Castellen et al., 2011[3]), TnrA in *Bacillus subtilis *(Kayumov et al., 2011[13]) and AmtR in *Corynebacterium glutamicum *(Beckers et al., 2005[2]). The binding of PII allosteric effectors have been reported for PII in *Bacillus subtilis *(Kayumov et al., 2011[13]) and *Mycobacterium tuberculosis *(Bandyopadhyay et al., 2010[1]). To our knowledge, PII from Gram-positive bacteria that have been purified and studied *in vitro *are from *B. subtilis, S. mutans *and *M. tuberculosis. *In all three cases, GlnK has been engineered to amend purification and therefore the physiological relevance of these studies are questionable as the binding of the PII effectors usually involve the very final extensions of the protein (Truan et al., 2010[22]; Chellamuthu et al., 2014[4]).

Here we describe a simple and reproducible protocol for the purification of untagged GlnK from the biotechnological and medical interesting organisms *Streptomyces coelicolor, Rhodococcus jostii *and* Mycobacterium tuberculosis. *This protocol will help to define the roles of GlnK in Gram-positive Actinobacteria.

## Material and Methods

### Protein expression

The GlnK coding sequences from *M. tuberculosis *H37Rv (GI - 15610056), *S. coelicolor* A3(2) (GI - 32141267) and *R. jostii *RHA1 (GI - 111023490) were codon optimized, commercially synthesized and cloned into the *Nde*I/*Bam*HI sites of the pET29a vector by GeneScript to express native proteins. From here on we will designate these proteins as MtGlnK, ScGlnK and RjGlnK. These constructs were introduced into *E. coli *BL21 (λDE3), these cells were growth in 400 ml of LB containing kanamycin 50 μg.ml^-1^ at 37 °C, 200 rpm to an O.D_600 nm _of 0.5. Protein induction was achieved by adding 0.5 mM IPTG and culturing for 3 hours at the same conditions. Cells were collected by centrifugation and stored at -80 °C.

### Purification of M. tuberculosis and R. jostii GlnK

The cell pellets were resuspended in 20 ml of ice cold buffer 50 mM Tris-HCl pH 8, 100 mM KCl, 1 mM EDTA and 20 % (v/v) glycerol and lysed by sonication. The crude extracts were then kept in a heat treatment of 70 °C for 10 min followed by incubation on ice for 10 min as described (Moure et al., 2012[17]). The extracts were clarified by centrifugation at 30,000 g, 20 min at 4 °C, resulting in the soluble fraction 1 (SF1). The SF1 was precipitated with ammonium sulfate at 4 °C (final concentration of 700 mM) for 1 hour. The extracts were centrifuged at 30,000 g, 20 min at 4 °C, resulting in the SF2, which were loaded onto a 8 ml phenyl sepharose column (GE-healthcare) pre-equilibrated in the resuspension buffer containing 700 mM of ammonium sulfate. The column was washed with two column volumes of this buffer and proteins were eluted with 14 ml buffer stepwise decreasing the ammonium sulfate concentration (50 mM Tris-HCl pH 8 containing 630, 595, 560, 490, 385, 280 or 0 mM ammonium sulfate). Fractions of 1.5 ml were collected. The most pure and concentrated fractions were pooled in 13 ml fraction and loaded onto a Sephacryl S 200 column with a flow of 1 ml^.^min^-1^ using 50 mM Tris-HCl pH 8, 100 mM KCl and 5 % (v/v) glycerol as running buffer. Sephacryl S 200 was calibrated with a range of molecular mass standards (Sigma) (β-amilase, 200 kDa; albumin, 66 kDa; carbonic anhydrase, 29 kDa; cytochrome c, 12.4 kDa) (Figure S1). Proteins were stored at -80 °C.

### Purification of S. coelicolor GlnK

The cell pellet was resuspended in 20 ml of ice cold buffer 50 mM Tris-HCl pH 8, 10 mM KCl and 10 % (v/v) glycerol and lysed by sonication. The crude extract was clarified by centrifugation at 30,000 g, 20 min at 4 °C and the soluble fraction was loaded onto a 3 ml Q-sepharose FF column (GE-Healthcare) pre-equilibrated with the resuspension buffer. Proteins were eluted in resuspension buffer with a segmented gradient of increasing concentrations of KCl 50, 100, 200, 300, 400 and 500 mM. The most pure and concentrated fractions were pooled and loaded onto a Sephacryl S200 column using 50 mM Tris-HCl pH 8, 100 mM KCl and 5 % (v/v) glycerol as running buffer. The protein was stored at -80 °C.

### Protein quantification

Total protein concentration was determined using Bradford reagent (BioRad) according to the manufacturer's protocol. Bovine serum albumin (BSA) was used to generate a standard curve.

### Circular dichroism (CD) and melting temperature determination

CD analyses were performed on a JASCO J-815 (JAPAN Spectroscopy & Chromatography Technology) spectropolarimeter coupled to a temperature controller. Proteins were diluted in 20 mM TrisHCl pH 8 to final trimer concentration of 0.5 µM. The CD spectra were recorded at 25 °C in the measure range of 195-300 nm. The baseline was corrected by subtracting the buffer spectrum. The secondary structure content was calculated using K2D3 software (Louis-Jeune et al., 2012[15]).

The Tm (midpoint of thermal denaturation) was calculated by measuring the CD signal at 222 nm with increasing temperature in a continuous ramp mode (1 °C/min). The temperature was allowed to increase from 30 up to 100 °C. The fraction of folded protein was calculated assuming that the CD signal at 222 nm 30 °C corresponds to fully folded protein and that the CD signal of the unfolded protein varies linearly with temperature. The Tm was determined applying a first derivative graphic using GraphPrism 6.0. To verify if the protein was able to refold after thermal denaturation, the temperature was reduced from 100 to 30 °C (-1 °C/min) and the CD signal at 222 nm was monitored.

## Results and Discussion

### Purification of MtGlnK and RjGlnK

Untagged recombinant GlnK from three Gram-positive bacteria *M. tuberculosis, S. coelicolor *and *R. jostii* (MtGlnK, RjGlnK and ScGlnK) were expressed in *E. coli *BL21 (DE3), all three proteins were present in high amounts in the soluble fraction of the cell extracts (data not shown). Initial screening of binding of MtGlnK to different matrices (Q-sepharose, CM-sepharose, DEAE-sepharose and Heparin) was conducted. However, MtGlnK showed little or weak interaction with all matrices tested (not shown). We noticed that MtGlnK remains soluble in ammonium sulfate up to 50 % saturation. Hence, we tested the combination of ammonium sulfate treatment followed by hydrophobic interaction using a Phenyl sepharose column. Even though this procedure generated promising results, the MtGlnK preparation still remained with significant amount of contaminants when analyzed by SDS-PAGE (not shown). 

We showed previously that GlnK from different Proteobacteria are stable at temperatures up to 80 °C. This property has been previously exploited to facilitated protein purification by the introduction of a thermal treatment step which results in the precipitation of contaminants while keeping active GlnK in the soluble fraction (Moure et al., 2012[17]). In order to verify if thermal resistance is a characteristic shared by Actinobacteria GlnK, extracts of cells expressing each protein (MtGlnK, RjGlnK and ScGlnK) were incubated at 70 °C for 10 min followed by incubation on ice for 10 min. The extracts were clarified by centrifugation and the presence of GlnK in the soluble and precipitated fractions were analyzed. SDS-PAGE analysis showed that while MtGlnK and RjGlnK remained fully soluble after the thermal treatment, in contrast, nearly all ScGlnK precipitated (not shown). These data suggest that MtGlnK and RjGlnK are resistant to heat while ScGlnK is not. 

**Figure 1:**
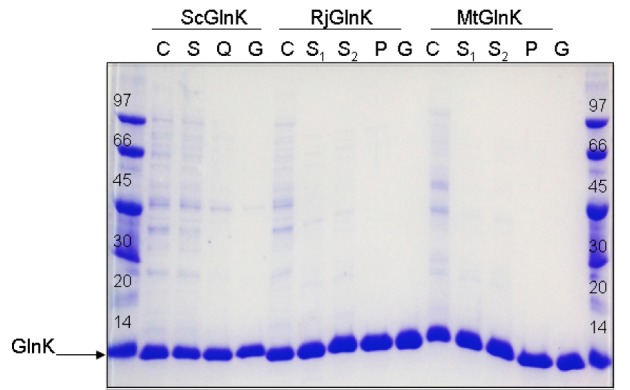
Analysis of the purification steps of the GlnK proteins. 5 μg of each sample was loaded on Tricine-SDS-PAGE. C - crude extract; S - Soluble fraction; Q - after Q sepharose; G - after gel filtration; S_1 _- soluble fraction after thermal treatment; S_2 _- soluble fraction after ammonium sulfate; P - after phenyl sepharose. The arrow indicates the GlnK protein. Molecular weight markers (kDa) were applied in the first and final lanes.

**Figure 2:**
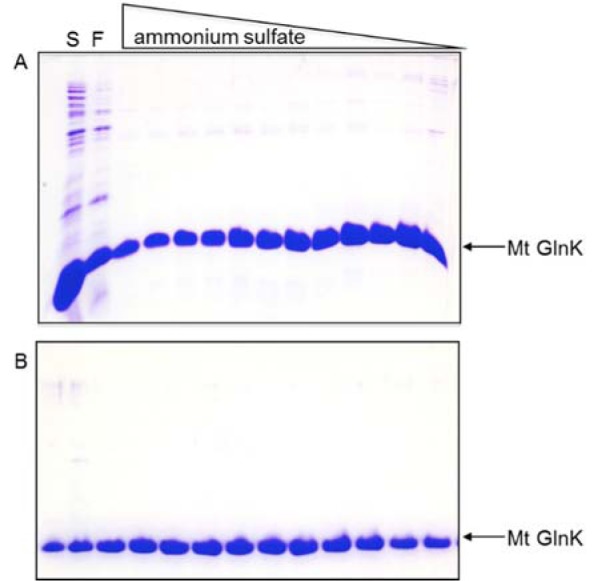
Purification profile of MtGlnK. Tricine-SDS-PAGE analysis of: (A) Fractions eluted from Phenyl sepharose column by decreasing the ammonium sulfate concentration. (B) Fractions eluted from gel filtration Sephacryl S200 column. S - Soluble fraction; F - Flow through.

**Figure 3:**
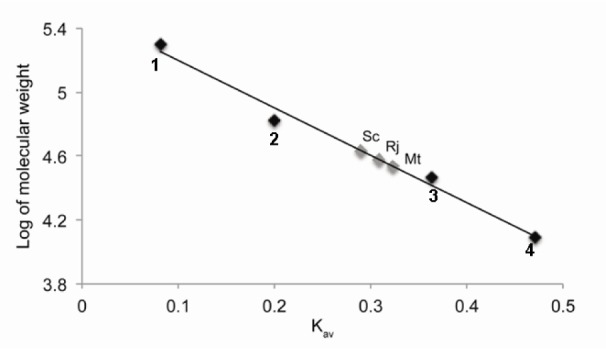
Gel filtration analysis of GlnK proteins. Gel filtration was performed on a Sephacryl S200 HR 26/60 column in 50 mM Tris-HCl pH 8, 100 mM KCl and 5 % (v/v) glycerol. The column was calibrated with a range of molecular mass standards (Sigma). 1, β-amilase, 200 kDa; 2, albumin, 66 kDa; 3, carbonic anhydrase, 29 kDa; 4, cytochrome c, 12.4 kDa. According to the standard curve, the estimated molecular weight for ScGlnK, RjGlnK and MtGlnK proteins were 43, 38 and 34 kDa, respectively.

In the final MtGlnK purification protocol, we applied a thermal treatment step (70 °C for 10 min, followed by 10 min on ice) in the cell extract leading to a significantly reduction in the levels of contaminants (Figure 1(Fig. 1), compare C and S1). The soluble fraction (S1) was incubated with ammonium sulfate 30 % saturation and the soluble fraction was loaded onto a Phenyl sepharose column. MtGlnK was eluted by decreasing the ammonium sulfate concentration (Figure 2A(Fig. 2)), the most pure and concentrated fractions were pooled fractionated onto a Sephacryl S200 column (Figure 2B(Fig. 2)). MtGlnK eluted as a single symmetric peak with elution volume corresponding to 34 kDa (Figure 3(Fig. 3)), which fits the calculated mass for the trimer (36.6 kDa). 

The purification protocol allowed reproducible purification of MtGlnK (> 99 % pure) in high yields (Figure 1(Fig. 1) and Table 1(Tab. 1)). This very same protocol was successfully applied for RjGlnK (Figure 1(Fig. 1), Table 1(Tab. 1) and Figure S1). The elution volume after gel filtration of RjGlnK corresponded to 38 kDa, which fits with the calculated mass for the trimer (36.2 kDa). 

### Purification of ScGlnK 

**Figure 4:**
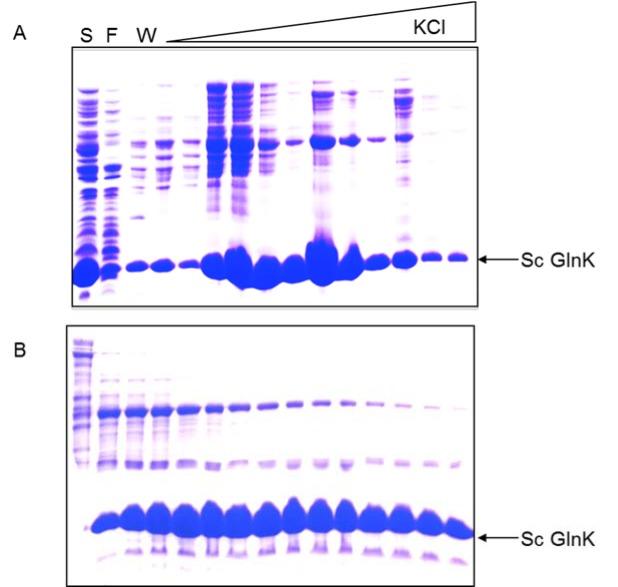
Purification profile of ScGlnK. Tricine-SDS-PAGE analysis of: (A) Fractions eluted from Q sepharose column by increasing the KCl concentration. (B) Fractions eluted from gel filtration Sephacryl S200 column. S - Soluble fraction; F - Flow through; W - Washing fraction.

The thermal treatment could not be applied for ScGlnK as the protein was completely insoluble after heating the extract (see above). Furthermore, ammonium sulfate fractionation was not effective because ScGlnK precipitated with small amounts of ammonium sulfate (not shown). We tested the ability of ScGlnK to interact with the matrices Heparin sepharose, DEAE sepharose and Q sepharose (GE Healthcare) (not shown). ScGlnK showed weak interaction with Heparin and DEAE sepharose, and stronger interaction with Q sepharose (not shown). In the final ScGlnK purification protocol, supernatant of cell extract was loaded onto a Q sepharose column (GE healthcare) and eluted with increasing KCl concentration (Figure 4A(Fig. 4)). The most concentrated fractions were pooled and fractionated onto a Sephacryl S200 gel filtration column (Figure 4B(Fig. 4)). ScGlnK eluted as a trimer, in a single peak corresponding to 43 kDa (Figure 3(Fig. 3)), which fits with calculated mass for ScGlnK trimer (36.7kDa). The purification protocol was efficient and resulted in a protein 95 % pure, in high yields (Figure 1(Fig. 1) and Table 1(Tab. 1)).

### Circular dichroism analysis and Tm determination

**Figure 5:**
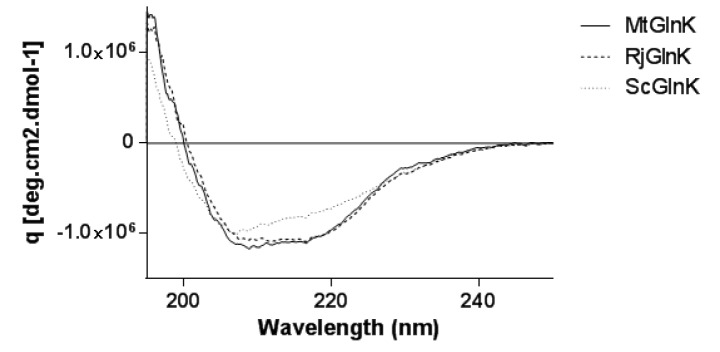
CD spectrum of MtGlnK, ScGlnK and RjGlnK. CD was recorded at 30°C. The secondary structure content was calculated using K2D3 software.

The secondary structures of the purified proteins were analyzed by circular dichroism (CD). All proteins present CD spectra of typically folded protein (Figure 5(Fig. 5)) containing both α-helix and β-sheets as expected based on the known structures of the PII family (MtGlnK α 39 % β 15 %, RjGlnK α 38 % β 16 %, ScGlnK α 34 % β 16 %). However, while MtGlnK and RjGlnK exhibited very similar CD spectra, ScGlnK showed a slightly different profile near 230 nm which could reflect slightly different structures.

**Figure 6:**
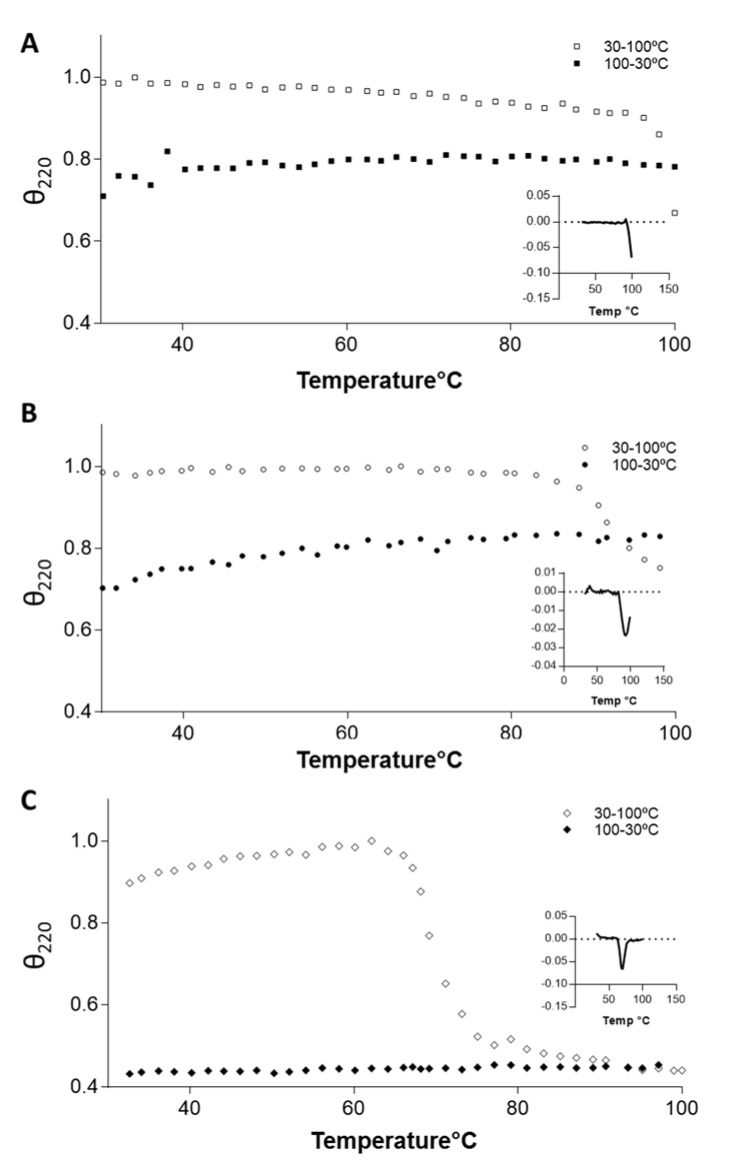
Thermal CD profiles of RjGlnK (A), MtGlnK (B) and ScGlnK (C). Thermal shifts of GlnK were monitored by CD analysis. Change in ellipticity at 222 nm (θ_222_) at the temperature ramp from 30 to 100º C (empty squares) and from 100 to 30º C (black squares). Insert shows the first derivative graphic obtained from the data, the bottom of the valley is used to determine the Tm.

We used CD analysis to determine the melting temperature (Tm) of MtGlnK, RjGlnK and ScGlnK. MtGlnK presented a Tm of 91.3 °C (Figure 6B(Fig. 6)) and RjGlnK a Tm >100 °C (Figure 6A(Fig. 6)). In contrast, the Tm of ScGlnK was much lower (69 °C; Figure 6C(Fig. 6)) and all the ScGlnK that denatured upon exposure to 100 °C could not regain proper folding after returning to 30 °C, as observed by the flat line in the 100 to 30 °C ramp (Figure 6C(Fig. 6) black diamonds). These results validate the use of a thermal treatment step for MtGlnK and RjGlnK purification and explained why ScGlnK fully precipitated in our attempts to use the thermal treatment for ScGlnK purification.

## Conclusions

Here we describe a simple, reproducible and efficient protocol for the purification of untagged GlnK from three different Actinobacteria. Protein folding was accessed after purification using gel filtration chromatography and CD analysis, both showed that we recovered properly folded proteins. Interestingly, ScGlnK showed different physicochemical properties from the Mt and Rj orthologues regarding its CD spectrum (Figure 5(Fig. 5)), solubility in ammonium sulfate and thermal stability (Figure 6(Fig. 6)). These different physicochemical properties are supported by sequence analysis showing that ScGlnK is more distant related to MtGlnK and RjGlnK ortologues (Figure S2). Deeper structural analysis will be required to explain the molecular basis of such differences. The purification protocol described here will facilitate biochemical analysis and help to uncover novel functions of the multitasking PII proteins from Actinobacteria species of medical and biotechnological importance.

## Acknowledgements

We are grateful to Marcela V. Medeiros_,_ Heloisa B.S. Sanchuki for helping in the initial phase of this project and Adrian R. S. dos Santos for constructing the GlnK phylogenetic tree. Valter de Baura and Roseli Prado are acknowledged for their technical support. This work was supported by INCT, CNPq, CAPES and Fundação Araucária.

## Supplementary Material

Supplementary information

## Figures and Tables

**Table 1:**
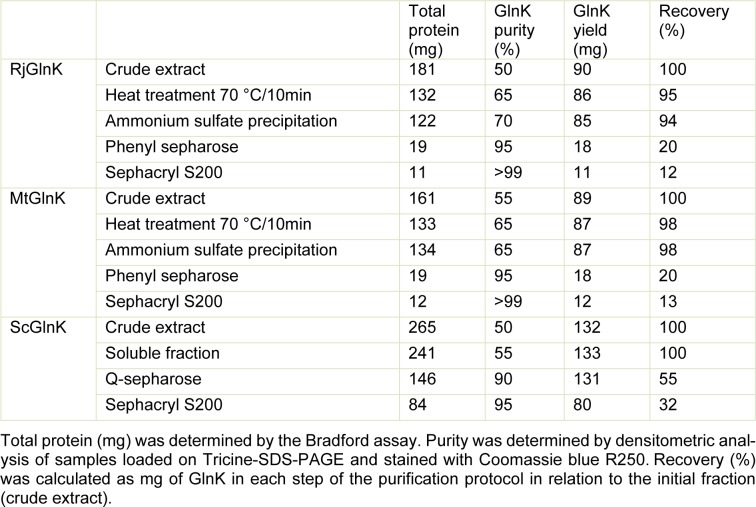
Purification of Actinobacteria GlnK proteins
