# Supplementary material for: Expression and purification of untagged GlnK proteins from actinobacteria
Source: EXCLI J. 2017 Jun 27;16:949–58. doi: 10.17179/excli2017-394 (PMC5579400; doi:10.17179/excli2017-394)
Supplement: Supplementary information [file EXCLI-16-949-s-001.pdf]

EXCLI Journal 2017;16:949-958 – ISSN 1611-2156
Received: April 27, 2017, accepted: June 12, 2017, published: June 27, 2017

Supplementary information to:

EXPRESSION AND PURIFICATION OF UNTAGGED GLNK PROTEINS FROM ACTINOBACTERIA

Edileusa C.M. Gerhardt[1], Vivian R. Moure[1], Andrey W. Souza[1], Fabio O. Pedrosa[1], Emanuel M. Souza[1], Lautaro Diacovich[2], Hugo Gramajo[2], Luciano F. Huergo[1,3*]

[1] Departamento de Bioquímica e Biologia Molecular, UFPR, Curitiba, Brazil
[2] Instituto de Biologıa Molecular y Celular de Rosario (IBR-CONICET), Facultad de Ciencias Bioquımicas y Farmaceuticas, Universidad Nacional de Rosario, Rosario, Argentina
[3] Setor Litoral, UFPR, Matinhos, Brazil

* Corresponding author: Prof Luciano F. Huergo – Universidade Federal do Paraná, Setor Litoral, Rua Jaguariaíva, 512, Caiobá - Matinhos - Paraná – Brazil, CEP: 83260-000, Phone +55 41 96765856, +55 41 35118321, +55 41 35118393;
E-mail: Luciano.huergo@gmail.com

http://dx.doi.org/10.17179/excli2017-394

This is an Open Access article distributed under the terms of the Creative Commons Attribution License (http://creativecommons.org/licenses/by/4.0/).

EXCLI Journal 2017;16:949-958 – ISSN 1611-2156
Received: April 27, 2017, accepted: June 12, 2017, published: June 27, 2017

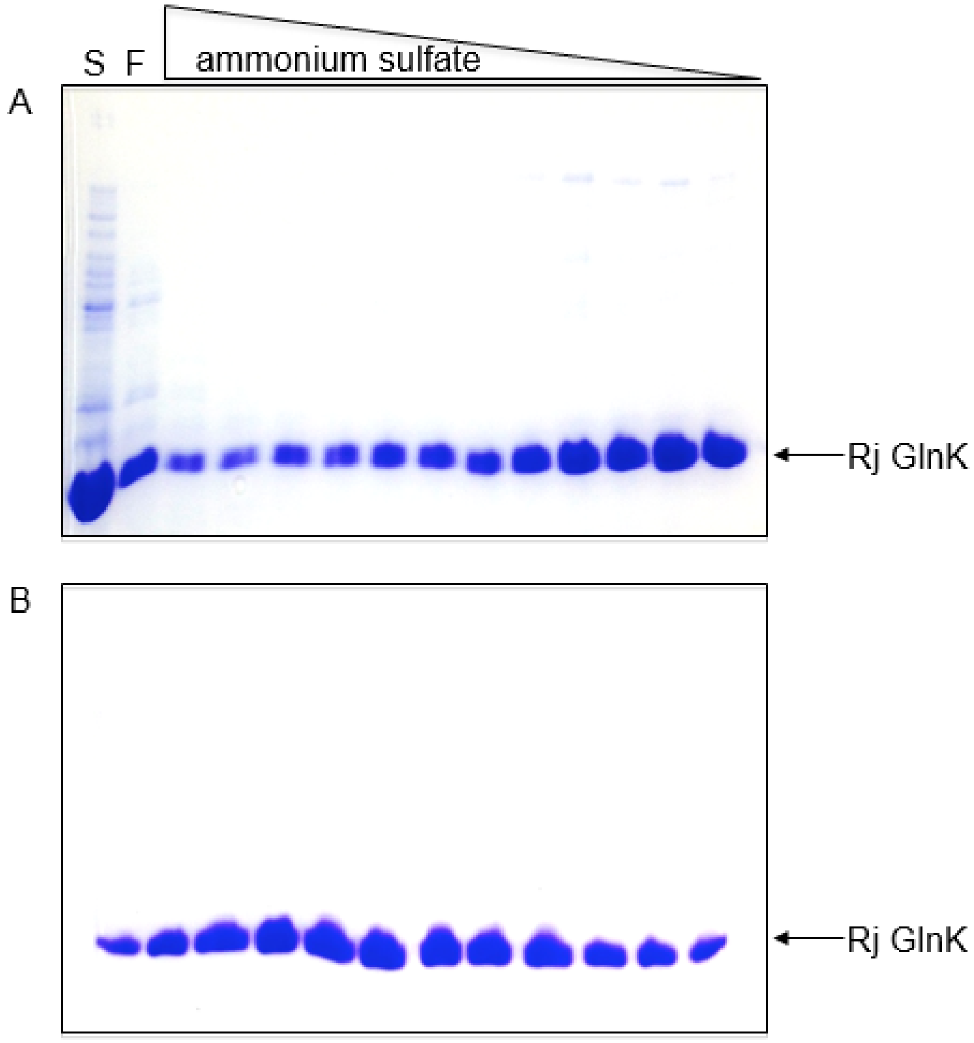

Figure S1: Purification profile of RjGlnK. Tricine-SDS-PAGE analysis of (**A**) Fractions eluted from Phenyl sepharose column by decreasing the ammonium sulfate concentration. (**B**) Fractions eluted from gel filtration Sephacryl S200. S – Soluble fraction; F – Flow through.

EXCLI Journal 2017;16:949-958 – ISSN 1611-2156
Received: April 27, 2017, accepted: June 12, 2017, published: June 27, 2017

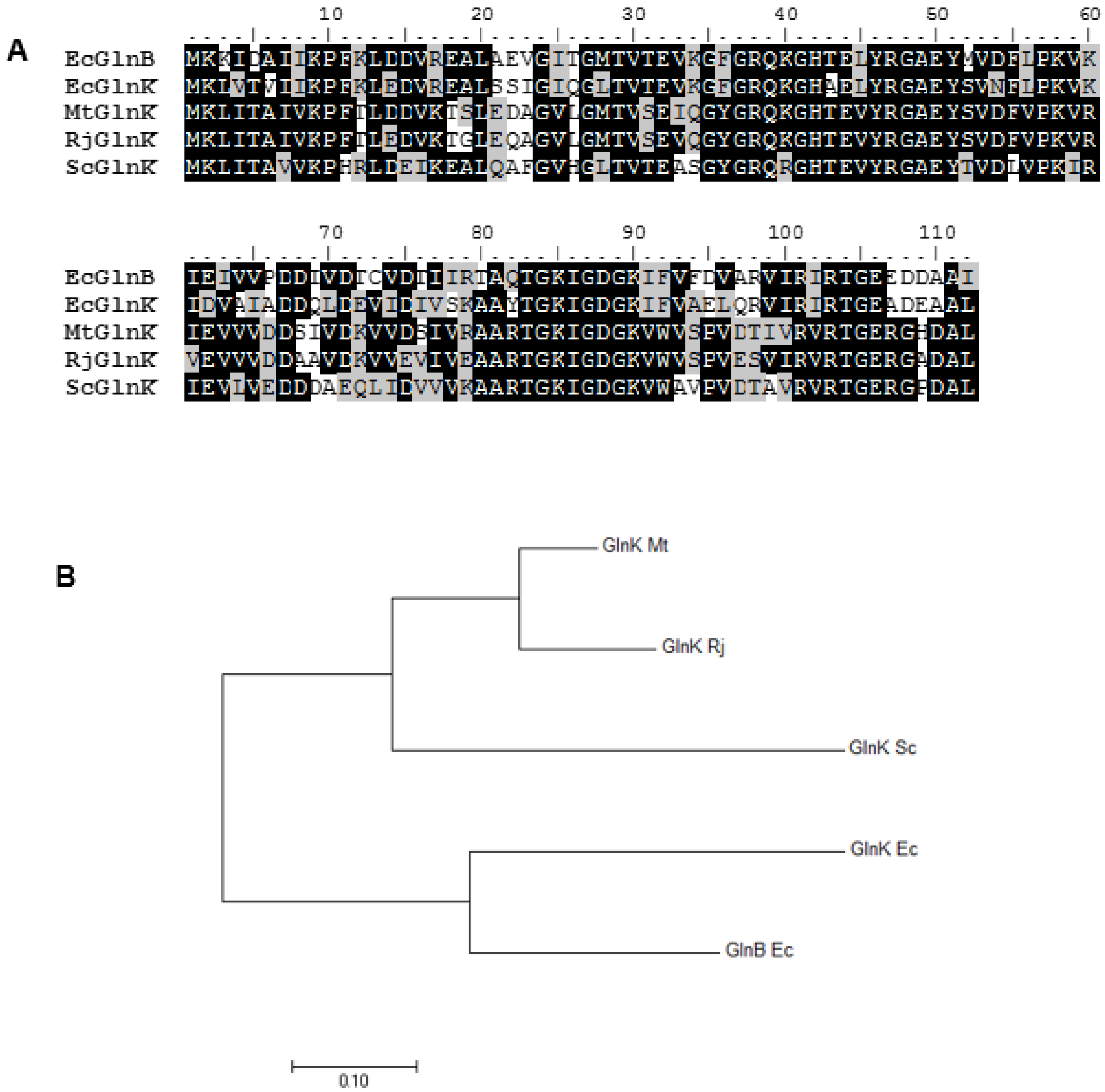

Figure S2: (**A**) Clustal W alignment of PII proteins from *E. coli*, *M. tuberculosis*, *R. jostii* and *S. coelicolor*. Identical amino acids are shown in black and similar in grey. (**B**) Molecular Phylogenetic analysis by the Maximum Likelihood method using MEGA7. The tree is drawn to scale, with branch lengths measured in the number of substitutions per site.
